# Breaking barriers for glioblastoma with a path to enhanced drug delivery

**DOI:** 10.1038/s41467-023-41694-9

**Published:** 2023-09-22

**Authors:** Imran Noorani, Jorge de la Rosa

**Affiliations:** 1 Cancer Evolution and Genome Instability Laboratory, The Francis Crick Institute and University College London, London, UK; 2 Department of Neurosurgery, National Hospital for Neurology and Neurosurgery, London, UK; 3 Department of Medicine, University of Cambridge School of Clinical Medicine, University of Cambridge, Cambridge Biomedical Campus, Cambridge, CB2 0QQ UK

**Keywords:** CNS cancer, Cancer models, Cancer therapy, Drug delivery

## Abstract

Progress in treatment for glioblastoma is hindered by the blood-brain barrier (BBB). In genetic mouse models recapitulating brain invasion and abnormal angiogenesis of human glioblastoma, Cai and colleagues demonstrate that optical modulation of the BBB with nanoparticles boosts intratumoural chemotherapy concentration, prolonging survival. We discuss prospects for clinical translation of exemplary innovative techniques.

## Challenges with drug delivery for brain cancer

There are several challenges facing the treatment of brain cancer. Gliomas are the most common primary intrinsic brain tumours. Glioblastoma, or a high-grade glioma, is a type of brain cancer that has a devastating prognosis, and for which oncogene-targeted and immune-targeted therapies have yet to make a significant impact in the clinic^1^. The standard of care remains maximal safe surgical resection, followed by radiotherapy and temozolomide chemotherapy^2^. One of the most important hurdles to overcome to improve patient outcomes in glioblastoma is to enable therapies to pass through the blood–brain barrier (BBB) and reach the tumour directly for cancer cell killing.

The BBB is made up of microvascular endothelial cells separating blood from brain interstitial fluid, while communicating with brain cells such as astrocytes and pericytes. The BBB is strengthened by tight and adherens junctions between neighbouring endothelial cells, creating a small pore diameter (1.4–1.8 nm) through which only limited particles of a small size can pass. Indeed, around 98% of small molecules and almost all biological macromolecular agents (e.g., antibodies and growth factors) are blocked from passing through the BBB, severely hampering drug delivery to the brain^3^. Furthermore, delivery to brain cancer specifically has nuanced issues: glioblastomas are heterogeneous cancers from a molecular and histopathological perspective, such that some areas of a tumour have BBB breakdown and increased permeability, whereas others do not. Additionally, the invasive margins of glioblastoma are composed of normal brain tissue infiltrated by cancer cells. In this region, the BBB remains intact, restricting drug delivery to invasive margins that have a unique immune microenvironment and where tumour recurrences are most likely to occur after treatment^4^.

**Fig. 1:**
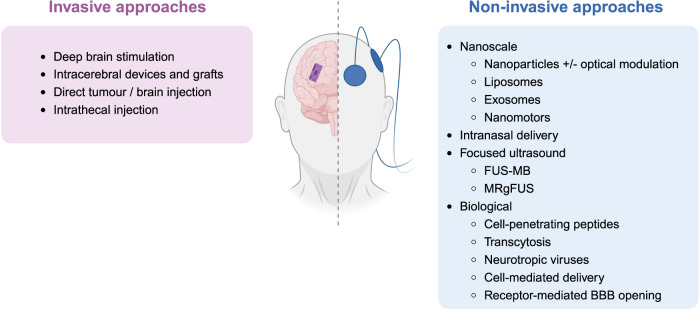
Technologies for delivering therapeutics to the brain, particularly for brain cancer treatment, can be categorised as either invasive or non-invasive. Emerging among these approaches are nanoscale technologies that serve as experimental tools for circumventing the BBB to facilitate drug delivery into glioblastoma. FUS-MB focused ultrasound, with intravenous administration of microbubbles; MRgFUS MRI-guided focused ultrasound^5^.

A plethora of techniques is being developed to improve BBB penetration in glioblastoma (Fig. 1). These techniques include hyperosmolar agents (such as mannitol), tight junction-modulating agents to open tight junctions, inhibitors of drug efflux transporters, and receptor-mediated transcytosis. Another method that is undergoing testing in early-phase clinical trials for BBB opening is focused ultrasound with intravenous microbubbles;^5^ a recent advancement in this method is MRI acoustic emission-controlled focused ultrasound^6^. However, novel technologies for BBB opening are critical to ensure maximal drug penetration and minimal toxicity in brain cancer treatment.

While new methods are being explored to improve drug delivery across the BBB, mouse models remain essential tools for preclinical testing of potential chemotherapies for glioblastoma before advancing to clinical trials^7^. While in vitro systems offer some advantages such as being easier to implement, faster and more cost-effective, in vivo cancer models are essential for recapitulating the three-dimensional organisation of tumours and their interactions within the intricate tumour microenvironment and host response. In this context, glioblastoma mouse models provide a more physiologically relevant setting to study cancer behaviour and drug responses. Several types of brain cancer mouse models exist, including syngeneic transplantation of mutated neural or cancer cells, genetically engineered mouse models (GEMMs), and patient-derived xenografts^8^. Different models have their own strengths and weaknesses, but a common issue is the inability to fully replicate cancer invasion into the surrounding brain and the aberrant microvasculature of glioblastoma.

Now, Cai et al. introduce an alternative technique to enhance drug delivery into brain tumours, employing optical nanoparticle-based modulation of the BBB. In their study, published in *Nature Communications*, the authors developed two transplantable glioblastoma mouse models that reflect significant histopathological features observed in patient cancers^9^. They subsequently demonstrate that optical modulation of the BBB using tight junction-targeted gold nanoparticles can significantly enhance the delivery of the chemotherapeutic drug Taxol into brain cancers. Importantly, this intervention resulted in improved survival rates in mice.

## Optical BBB modulation enhances glioblastoma drug delivery in mice

**Fig. 2:**
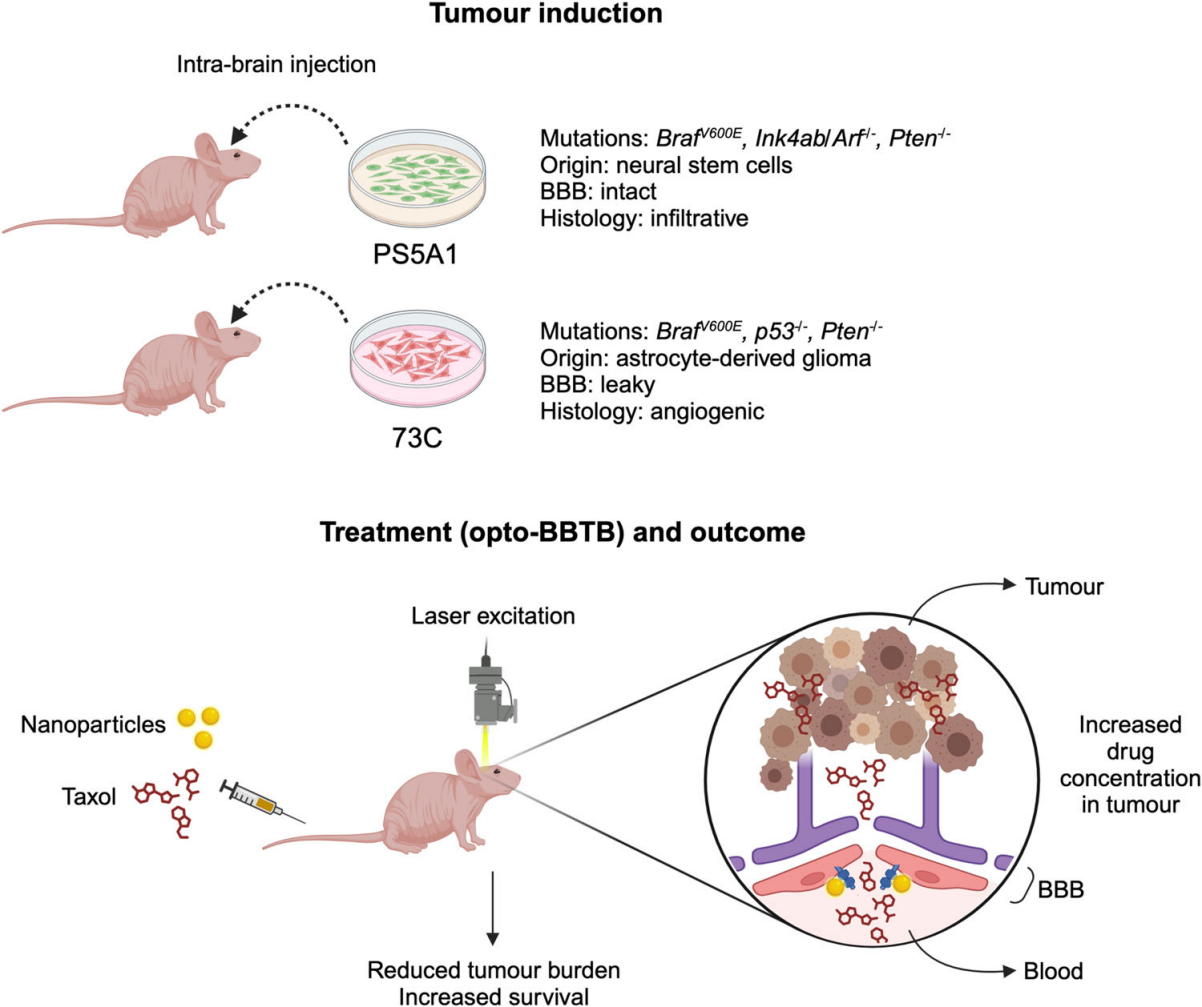
Transplantable glioblastoma mouse models and therapeutic strategy. Top: tumour induction by intra-brain injection of two distinct glioblastoma cell lines. Genetic and biological characteristics of these cell lines are highlighted. Bottom: application of “optical blood-brain-tumour barrier” (optoBBTB) modulation. Gold nanoparticles, targeted to JAM-A tight junction protein, are intravenously administered alongside Taxol. A transcranial 532 nm picosecond laser activates nanoparticles within the tumour, temporarily weakening the blood-brain barrier (BBB) for enhanced drug delivery. The BBB is depicted by endothelial cells with tight junctions, and astrocytes. Pericytes are omitted for clarity.

While numerous mouse models of glioblastoma are employed in preclinical research, many fail to fully replicate the genomic or clinicopathological characteristics of human glioblastoma. Cai et al. genetically engineered two mouse brain cancer cell lines and implanted them into the mouse cortex (Fig. 2). These cell lines encompass distinct mutations, with one carrying *Braf*^*V600E*^*, Ink4ab/Arf*^*-/-*^*, Pten*^*-/-*^ mutations and the other featuring a different set of mutations (*Braf*^*V600E*^, *p53*^*-/-*^*, Pten*^*-/-*^). With the exception of the relatively infrequent oncogene *Braf* mutation observed in human high-grade gliomas, the loss of the other tumour suppressor genes is a frequent occurrence in patients’ tumours^10^. These mouse models accurately portray key clinicopathological aspects of human glioblastomas: one model displays diffuse single-cell infiltration of cancer cells throughout the brain parenchyma, while the second model exhibits highly aberrant tumour vasculature yet limited brain invasion. Together, these two models effectively capture important features seen in human glioblastomas.

In the subsequent phase, the authors assessed the technique of optical blood–brain-tumour barrier (optoBBTB) modulation utilising gold nanoparticles (Fig. 2). They chose paclitaxel (Taxol) as a representative chemotherapy drug, which is applied in clinical practice for various solid cancers but not for glioblastoma due to its poor brain penetration.

Gold nanoparticles with a diameter of 50 nm, designed to target the tight junction protein JAM-A, were intravenously administered to mice harbouring glioblastoma. Subsequently, a transcranial 532 nm picosecond laser was applied to the tumour region to activate the gold nanoparticles and induce temporary weakening of the BBB, likely through increased paracellular permeability related to calcium influx^11^. This weakening facilitated the delivery of Taxol into the tumour. The BBB modulation demonstrated several attributes: (1) it was region-specific, enhancing drug delivery exclusively to the tumour and not the rest of the brain; (2) it was reversible, as the BBB restored within a single day; and 3) it was repeatable, allowing for three rounds of chemotherapy with BBB modulation and drug delivery to the tumour on each occasion. Notably, this approach not only increased the concentration of Taxol within the tumours, but it also significantly prolonged the survival of mice in both the infiltrative and angiogenic glioblastoma models. This extension in survival time correlated with decreased tumour cell proliferation and increased cell death in the tumours of mice treated with opto-BBTB.

## Translation to human brain cancer in the clinic

One limitation of this model pertains to the superficial location of the glioblastomas, situated in the cerebral cortex directly adjacent to the skull’s surface. While certain brain tumours in human patients share a similar location, a significant number are positioned deeper within the brain tissue^12^. Therefore, it remains to be determined whether optoBBTB modulation can effectively facilitate drug delivery to tumours located at greater depths within the brain. Moreover, it is important to note that mice possess relatively thin skulls compared to humans. As such, parameters for transcranial laser stimulation through thicker human cranial bone will require optimisation for practical application in humans. Proposed strategies for addressing these challenges include the use of near-infrared light-absorbing nanoparticles (as light at this wavelength has greater tissue penetration), or the placement of optical fibres into the cancer surgical cavity, allowing the delivery of side-emitting light to and beyond the tumour margins in patients. Another recent experimental tool is the use of “See-Shells”, digitally designed, transparent polymer skulls that offer long-term optical access to the brain. Although these “See-Shells” have the potential for optical BBB modulation, their application has only been tested in mice so far^13^.

It is important to recognise the substantial size disparity between glioblastomas commonly observed in patients and those in mice. The translation process to the clinic must also account for the requirement of significantly more repeated chemotherapy doses in human patients. Furthermore, adaptations must consider the alterations induced in tumours due to extended radiation and chemotherapy regimens. Additionally, careful consideration should be directed towards the risk of immunological responses associated with long-term or repetitive treatment of the nanoparticles. Addressing these factors comprehensively will be imperative before successfully transitioning this approach into clinical practice.

To further validate the approach, diverse mouse models like GEMMs and patient-derived xenografts could be explored. While the study employed clinically relevant glioblastoma mouse models to evaluate optical BBB modulation, it remains essential to investigate this technique in glioblastoma models that harbour other common oncogenic drivers with clinical relevance. Examples of such drivers include *EGFR* amplification, *PDGFRA* amplification, or an *IDH1* mutation associated with a distinct glioma subtype^14,15^. Moreover, incorporating immunocompetent mice into the study would provide a more realistic scenario, as both the tumour behaviour as well as drug delivery and therapeutic efficacy could be influenced by intact immune responses.

Finally, while Taxol is a relevant drug for glioblastoma whose efficacy is limited due to the BBB, it would be similarly interesting to test other chemotherapeutic drugs and antibodies with similar limitations using this methodology. In particular, antibodies targeting immune checkpoints PD-1 and CTLA-4 (such as pembrolizumab and ipilimumab) have not shown major clinical benefits for glioblastoma and brain metastases, despite their remarkable success in peripheral tumours. This disparity may be attributed to BBB challenges, which could potentially be overcome by exploring methods like optoBBTB^16^. By exploring the potential of optoBBTB modulation, researchers can gain valuable insights into enhancing drug delivery to the brain and improving the effectiveness of various treatments for glioblastoma and other brain cancers.

## Concluding remarks

Bypassing the BBB to enhance drug concentrations within tumours has long been a formidable challenge in the field of brain cancer therapy. Alongside the development of innovative methods to facilitate drug penetration into the brain, it remains crucial to advance the preclinical modelling of this disease. Such advancements are essential to ensure that emerging tools and therapies can be effectively evaluated, optimising their clinical potential.

In essence, the study conducted by Cai et al. not only introduces mouse models that replicate the invasive and angiogenic characteristics of human glioblastoma but also convincingly demonstrates the effectiveness of optical BBB modulation using nanoparticles. This approach enhances the infiltration of chemotherapy agents into brain tumours, ultimately extending overall survival. Other promising nanoscale technologies for BBB penetration are emerging, such as synthetic protein nanoparticles that leverage the BBB-penetrating capacity of natural proteins;^17^ BBB-penetrating bacteria loaded with photosensitive nanoparticles that destroy themselves and adjacent tumour cells upon laser irradiation (‘Trojan bacteria’)^18^; and nanomotors targeting brain endothelial cells, relying on glioblastoma microenvironment chemoattractants to infiltrate the tumour and directly deliver chemotherapy^19^. While promising, future endeavours must address challenges associated with the clinical application of optoBBTB and similar technologies, particularly the anatomical disparities between humans and mice. Nevertheless, this study, alongside recent advancements in BBB modulation techniques, holds great promise for enabling a new generation of targeted therapies, including oncogene-targeted and immune-based drugs, to effectively penetrate brain cancers.

## References

[1] White K (2020). New hints towards a precision medicine strategy for IDH wild-type glioblastoma. Ann. Oncol..

[2] Stupp R (2005). Radiotherapy plus concomitant and adjuvant temozolomide for glioblastoma. N. Engl. J. Med..

[3] Wu D (2023). The blood-brain barrier: structure, regulation, and drug delivery. Signal Transduct. Target Ther..

[4] Noorani I (2023). Clinical impact of anti-inflammatory microglia and macrophage phenotypes at glioblastoma margins. Brain Commun..

[5] Terstappen GC, Meyer AH, Bell RD, Zhang W (2021). Strategies for delivering therapeutics across the blood-brain barrier. Nat. Rev. Drug Discov..

[6] Anastasiadis P (2021). Localized blood-brain barrier opening in infiltrating gliomas with MRI-guided acoustic emissions-controlled focused ultrasound. Proc. Natl Acad. Sci. USA.

[7] Noorani I, Mischel PS, Swanton C (2022). Leveraging extrachromosomal DNA to fine-tune trials of targeted therapy for glioblastoma: opportunities and challenges. Nat. Rev. Clin. Oncol..

[8] Noorani I (2019). Genetically engineered mouse models of gliomas: technological developments for translational discoveries. Cancers (Basel)..

[9] Cai Q (2023). Optical blood-brain-tumor barrier modulation expands therapeutic options for glioblastoma treatment. Nat. Commun..

[10] Ceccarelli M (2016). Molecular profiling reveals biologically discrete subsets and pathways of progression in diffuse glioma. Cell.

[11] Li X (2023). Mechanobiological modulation of blood-brain barrier permeability by laser stimulation of endothelial-targeted nanoparticles. Nanoscale.

[12] Larjavaara S (2007). Incidence of gliomas by anatomic location. Neuro Oncol..

[13] Ghanbari L (2019). Cortex-wide neural interfacing via transparent polymer skulls. Nat. Commun..

[14] Noorani I (2020). PiggyBac mutagenesis and exome sequencing identify genetic driver landscapes and potential therapeutic targets of EGFR-mutant gliomas. Genome Biol..

[15] Berger TR, Wen PY, Lang-Orsini M, Chukwueke UN (2022). World Health Organization 2021 classification of central nervous system tumors and implications for therapy for adult-type gliomas: a review. JAMA Oncol..

[16] Edavettal S (2022). Enhanced delivery of antibodies across the blood-brain barrier via TEMs with inherent receptor-mediated phagocytosis. Med.

[17] Gregory JV (2020). Systemic brain tumor delivery of synthetic protein nanoparticles for glioblastoma therapy. Nat. Commun..

[18] Sun R (2022). Bacteria loaded with glucose polymer and photosensitive ICG silicon-nanoparticles for glioblastoma photothermal immunotherapy. Nat. Commun..

[19] Chen H (2023). A nitric-oxide driven chemotactic nanomotor for enhanced immunotherapy of glioblastoma. Nat. Commun..

